# Activation of TRPA1 in Bladder Suburothelial Myofibroblasts Counteracts TGF-β1-Induced Fibrotic Changes

**DOI:** 10.3390/ijms24119501

**Published:** 2023-05-30

**Authors:** Mengmeng Zhao, Ning Ding, Haoyu Wang, Shulu Zu, Hanwen Liu, Jiliang Wen, Jiaxin Liu, Nan Ge, Wenzhen Wang, Xiulin Zhang

**Affiliations:** Department of Urology, The Second Hospital of Shandong University, Jinan 250033, China

**Keywords:** bladder fibrosis, TRPA1 channel, TGF-β1, myofibroblasts, allylisothiocyanate, SCI

## Abstract

The activation of the transient receptor potential ankyrin 1 (TRPA1) channel has anti-fibrotic effects in the lung and intestine. Suburothelial myofibroblasts (subu−MyoFBs), a specialized subset of fibroblasts in the bladder, are known to express TRPA1. However, the role of the TRPA1 in the development of bladder fibrosis remains elusive. In this study, we use the transforming growth factor-β1 (TGF-β1) to induce fibrotic changes in subu−MyoFBs and assess the consequences of TRPA1 activation utilizing RT-qPCR, western blotting, and immunocytochemistry. TGF-β1 stimulation increased α-SMA, collagen type I alpha 1 chain(col1A1), collagen type III (col III), and fibronectin expression, while simultaneously suppressing TRPA1 in cultured human subu−MyoFBs. The activation of TRPA1, with its specific agonist allylisothiocyanate (AITC), inhibited TGF-β1-induced fibrotic changes, and part of these inhibition effects could be reversed by the TRPA1 antagonist, HC030031, or by reducing TRPA1 expression via RNA interference. Furthermore, AITC reduced spinal cord injury-induced fibrotic bladder changes in a rat model. The increased expression of TGF-β1, α-SMA, col1A1 and col III, and fibronectin, and the downregulation of TRPA1, were also detected in the mucosa of fibrotic human bladders. These findings suggest that TRPA1 plays a pivotal role in bladder fibrosis, and the negative cross talk between TRPA1 and TGF-β1 signaling may represent one of the mechanisms underlying fibrotic bladder lesions.

## 1. Introduction

Bladder fibrosis is a late-stage presentation of bladder outflow tract obstruction (BOO), spinal cord injury (SCI), radiation, or ketamine-induced cystitis [1,2,3]. Bladder fibrosis is characterized by the excessive deposition of the extracellular matrix (ECM), which is composed of collagen (type-I and -III), elastin, and fibronectin. ECM is secreted by the myofibroblasts for wound healing and tissue repair [1], which are the contractile phenotype of fibroblasts and are characterized by the expression of α-smooth muscle actin (α-SMA), a greater capacity for collagen secretion, and a resistance to apoptosis [4].

Transforming growth factor beta (TGF-β1) was reported to play a pivotal role in bladder fibrosis [5] and is known to promote the invasion, migration, and proliferation of myofibroblasts. It also increases the expression of α-SMA, and the synthesis of collagen. The importance of TGF-β1 was previously reported both in SCI [6] or BOO [7,8,9]-induced bladder fibrosis.

There is a growing interest in the role of transient receptor potential (TRP) channels and piezo1 channels in tissue fibrosis [10,11,12,13]. For instance, the TRPV4 channel was shown to mediate pulmonary fibrosis [10]. Piezo1 channels were shown to mediate renal fibrosis [12,13]. In contrast to the profibrotic effects of TRPV4 and piezo1, the TRPA1 channel was shown to exert anti-fibrotic effects in hepatic stellate cells [14], oral submucosa [15], lungs [16], and the intestine [17,18,19]. Critically, the activation of TRPA1 was shown to mediate the anti-fibrotic effects of pirfenidone [18], an approved antifibrotic drug for idiopathic pulmonary fibrosis. In addition, the TGF-β1-induced down-regulation of TRPA1 channels appeared to contribute to the resistance of human lung myofibroblasts to apoptosis [4]. However, the involvement of the TRPA1 channel in the development of bladder fibrosis has not been previously investigated.

Immediately below the bladder urothelium, there is a layer of interstitial cells (ICs). They exhibit the characteristics of myofibroblasts and express vimentin (Vim), a mesenchymal marker, and α-SMA, a marker of myogenic differentiation [20,21,22]. Such suburothelial myofibroblasts (subu−MyoFBs) were detected in several species, including humans [23,24]. Notably, a recent study demonstrated that Vim^+^ subu−MyoFBs in the wall of the mouse bladder exhibit all of the ultrastructural hallmarks of fibroblasts, and it was suggested that these Vim^+^ fibroblasts may contribute to pathological bladder remodeling and fibrosis [25].

It was also reported that the vast majority (>90%) of subu−MyoFBs express TRPA1 channels in humans, guinea pigs, and pigs [26,27]. However, the functional role of these TRPA1 channels at this cellular localization remains to be elucidated.

In the present study, we investigate the roles of subu−MyoFBs in bladder fibrosis and the potential involvement of the TRPA1 channels in this process. First, we establish an in vitro model of TGF-β1-induced profibrotic changes in cultured human subu−MyoFBs. Then, we investigate the effects of TRPA1 agonists on TGF-β1-induced profibrotic changes in cultured human subu−MyoFBs (in vitro) or in SCI model rats (in vivo). Finally, we investigate fibrotic changes and TRPA1 expression levels in the lamina propria of the bladder in patients with SCI.

## 2. Results

### 2.1. Bladder Subu-MyoFBs Became Pro-Fibrotic in Response to TGF-β1 Stimulation

**Figure 1:**
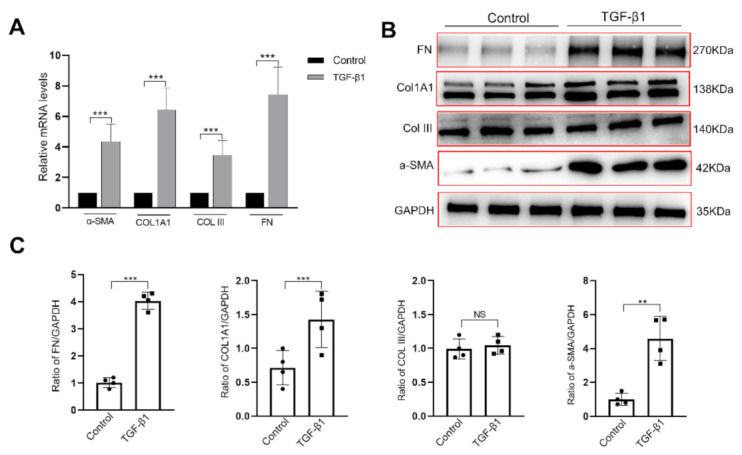
TGF-β1-induced fibrotic changes in bladder subu−MyoFBs. Cells were treated with 10 ng/mL of TGF-β1 for 24 h. (**A**) RT-qPCR analysis showing that the mRNA levels of *α-SMA*, collagen type I alpha 1 chain (*COL1A1*), collagen type III (*COLIII*) and fibronectin (*FN*) were upregulated in TGF-β1-treated cells. Data are expressed as fold-changes relative to *β-actin*. (**B**,**C**) Western blot results showing that the protein levels of α-SMA, COL1A1, and FN, but not COLIII, were increased in TGF-β1-treated cells. Data are presented as mean ± SEM (*n* = 4 experiments). NS: no significance. ** *p* < 0.01, and *** *p* < 0.001 compared with the control. *α-SMA*: α smooth muscle actin; *COL1A1*: collagen type I alpha 1 chain; *COLIII*: collagen type III; *FN*: fibronectin.

One recent study reported that PDGFR+ interstitial cells, including subu−MyoFBs, in the mouse bladder wall, represented specialized fibroblasts that may play a role in fibrotic changes in the bladder [25]. To explore whether this assumption was true in the human pathological processes, we examined human subu−MyoFBs responses to TGF-β1, the main profibrotic growth factor in cultured human subu−MyoFBs. As expected, after 24 h of incubation with TGF-β1 (10 ng/mL), subu−MyoFBs exhibited increased levels of mRNA and protein expression of *α-SMA* (*p* < 0.05, Figure 1A–C), a characteristic feature of profibrotic cells. Concomitantly, the cells also exhibited increased mRNA and protein expression levels of collagen type I alpha 1 chain (*COL1A1*) and fibronectin (*FN*) (*p* < 0.05, Figure 1A–C). While the collagen type III (*COLIII*) mRNA transcript levels were increased, its protein level was not changed after 24 h of TGF-β1 treatment.

### 2.2. TRPA1 Channels Are Abundantly Expressed in Bladder Subu-MyoFBs and Are Down-Regulated Following TGF-β1 Treatment

**Figure 2:**
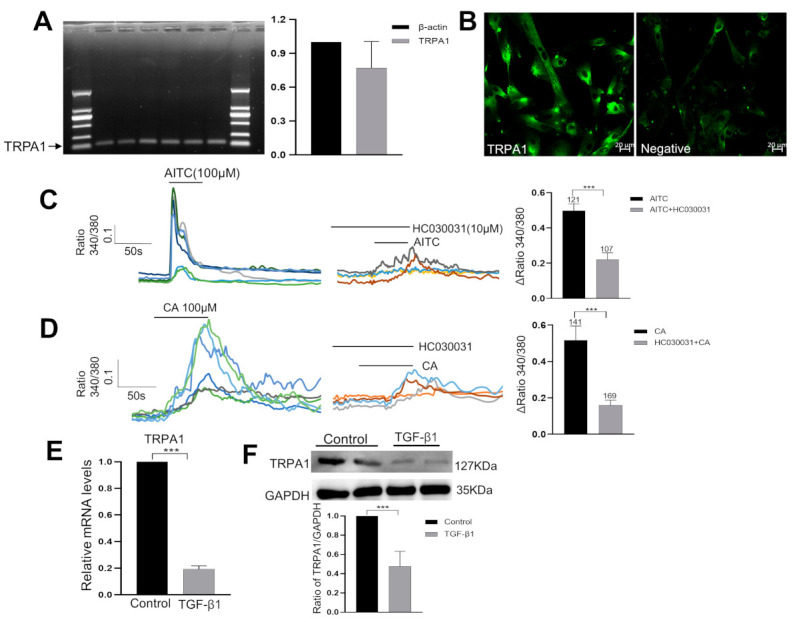
TRPA1 channels are expressed in bladder subu−MyoFBs and were down-regulated by TGF-β1. (**A**) The mRNA expression of *TRPA1*, as determined via RT-PCR (left). RT-qPCR analysis (right) revealed the relative expression levels of *TRPA1* to *β-actin* (*n* = 3 experiments). (**B**) Immunofluorescent results showing that TRPA1 protein was expressed in subu−MyoFBs and could be blocked by pretreatment with its blocking peptide. Scale bar = 20 µm. (**C**,**D**) Ca^2+^ imaging experiments showed that the specific TRPA1 agonists AITC (100 µM) and cinnamaldehyde (CA, 100 µM) elicited an increase in [Ca^2+^]_i_ in cultured myofibroblasts that could be blocked by the TRPA1 antagonist HC030031 (10 μM). Agonists were applied for 30–50 s, and HC030031 was applied 5 min prior to the agonists. Different colored lines indicate various cells. Bar figures represent summary data. The number above each bar indicates the cell number. *** *p* < 0.001. (**E**,**F**) TGF-β1 incubation (10 ng/mL for 24 h) significantly reduced the mRNA ((**E**), via RT-qPCR) and protein levels ((**F**), via WB) of TRPA1.

The expression of TRPA1 channels by human bladder subu−MyoFBs was previously reported in previously reports [26,27]. Consistent with these observations, we detected the expression of *TRPA1* at both the mRNA and protein level (Figure 2A,B). The functional activity of TRPA1 was demonstrated by Ca^2+^ imaging experiments (Figure 2C,D), where the TRPA1 agonists allylisothiocyanate (AITC, 100 μM) and cinnamaldehyde (CA, 100 μM) both induced a significant increase in the [Ca^2+^]_i_ in 65% (262 of 403) of the observed subu−MyoFBs. Furthermore, this Ca^2+^ influx was effectively blocked by the TRPA1 specific antagonist HC030031 (HC3, 10 μM) (Figure 2C,D).

It was also reported that TGF-β1 down-regulated TRPA1 expression on lung fibroblasts [4,16]. Consistent with those findings, we also found that the incubation of human subu−MyoFBs in the presence of TGF-β1 (10 ng/mL) for 24 h significantly reduced (*p* < 0.001) TRPA1 expression both at the mRNA (Figure 2E) and protein level (Figure 2F).

### 2.3. TRPA1 Agonists Suppressed TGF-β1-Induced Fibrotic Changes

**Figure 3:**
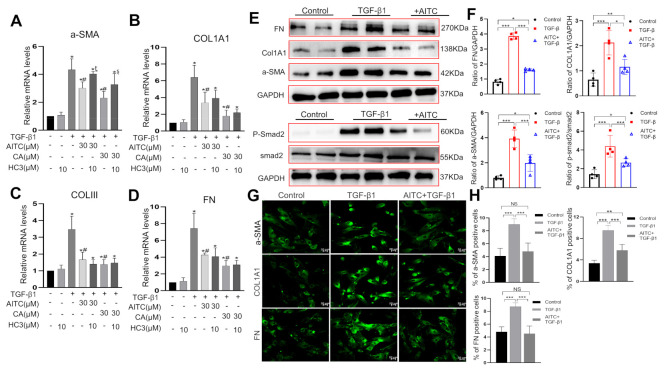
Activation of TRPA1 counteracted TGF-β1-induced fibrotic changes in bladder subu−MyoFBs. (**A**–**D**) RT-qPCR analysis showing that the application of the TRPA1 agonists AITC (30 µM) or cinnamaldehyde (CA, 30 µM) for 1 h prior to TGF-β1 treatment (10 ng/mL) significantly reduced the increase in mRNA expression of *α-SMA* (**A**), *COL1A1* (**B**), *COLIII* (**C**), and *FN* (**D**) induced by TGF-β1 stimulation. However, pretreatment with the TRPA1 antagonist HC030031 (HC3, 10 μM) 10 min prior to AITC or CA reversed the reduced effects of the TRPA1 agonist on *α-SMA*, but not on *col1A1*, *Col III* and *FN*. The summary data refer to four experiments. * *p* < 0.05 vs. control cells; # *p* < 0.05 vs. TGF-β1–treated cells; ȶ *p* < 0.05 vs. AITC+ TGF-treated cells; ȿ *p* < 0.05 vs. CA + TGF-treated cells. (**E**,**F**) Western blot analysis showing that pretreatment with the TRPA1 agonist AITC (30 µM) significantly reduced the increased protein expression of α-SMA, COL1A1, fibronectin, and phosphorylated Smad-2 (p-Smad2) induced by TGF-β1 stimulation. The figures on the right in F represent summary data from four experiments. To note, unlike α-SMA, COL1A1, and FN protein (which were measured 24 h after TGF-β1 treatment), p-Smad-2 was measured 2 h after TGF-β1 treatment (*n* = 4 experiments). (**G**,**H**) Immunostaining images of subu−MyoFBs showing that AITC pretreatment (right) reduced the increase in protein expression of α-SMA, COL1A1, and FN induced by TGF-β1 (middle). Target proteins are shown in green (**G**). Summary data for immunostaining images were analyzed using ImageJ software (**H**). AITC was administered 1 h prior to TGF-β1. * *p* < 0.05; ** *p* < 0.01; *** *p* < 0.001.

To investigate whether TRPA1 agonists could counteract TGF-β1-induced fibrotic changes in cultured human subu−MyoFBs, we administered the TRPA1 agonist AITC (30 μM) 1 h prior to TGF-β1 and examined the fibrotic changes 24 h after TGF-β1 treatment. We found that pretreatment with AITC significantly attenuated the TGF-β1-induced increase in the mRNA expression of *α-SMA*, collagens type I and III, and fibronectin (Figure 3A–D). Western blotting (Figure 3E,F) and immunofluorescent imaging (Figure 3G,H) further revealed the reduced protein expression levels of α-SMA, col1A1, and fibronectin. Interestingly, the anti-TGF-β1 effects of AITC could only be detected when AITC was administered prior to TGF-β1; these effects did not occur when the two agents were applied simultaneously. Another TRPA1 agonist, cinnamaldehyde (CA, 30 μM), also produced anti-TGF-β1 effects (Figure 3A–D). The administration of the TRPA1 antagonist HC030031 (HC3, 10 μM) partially blocked the anti-TGF-β1 effects of AITC; this blocking effect occurred for *α-SMA*, but not for collagens (type I and III) and fibronectin (Figure 3A–D).

The canonical downstream signal pathway of TGF-β1 involves the phosphorylation of Smad2/3 [28]. We also demonstrated TGF-β1-induced Smad2 phosphorylation in subu−MyoFBs (Figure 3E lower panel). Furthermore, Smad2 phosphorylation was significantly attenuated after AITC pretreatment, suggesting the involvement of this canonical pathway in the anti-fibrotic effects of AITC.

### 2.4. The Knockdown of TRPA1 Expression Reduced the Anti-TGF-β1 Effects of TRPA1 Agonists

**Figure 4:**
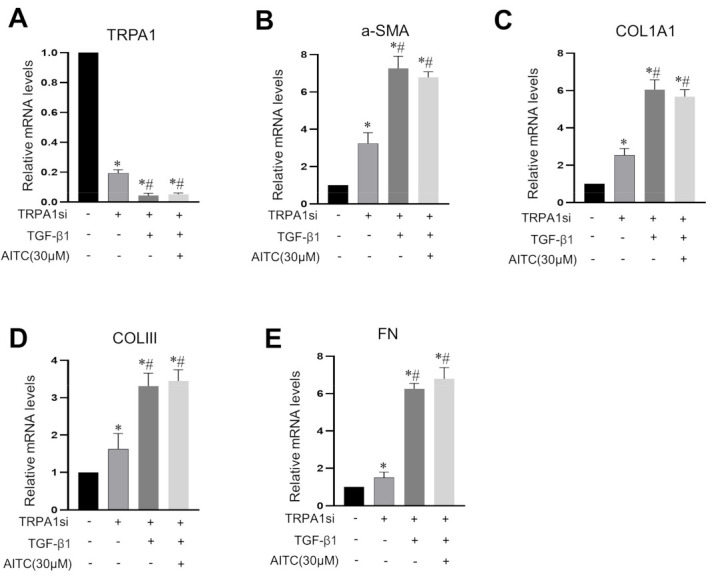
The knockdown of TRPA1 expression attenuated the anti−fibrotic effects of AITC. The relative mRNA expression levels of *TRPA1* (**A**), *α-SMA* (**B**), *COL1A1* (**C**), *COLIII* (**D**), and *FN* (**E**) were determined using real-time RT-qPCR. (**A**) Treatment with the anti-sense RNA of TRPA1 (TRPA1Si) reduced the mRNA expression of *TRPA1* to 19.4% and was further reduced to 4.3% via TGF-β1 treatment. However, the application of AITC did not exert any further impact on *TRPA1* expression. (**B**–**E**) The knockdown of *TRPA1* expression alone increased the mRNA expression levels of *α-SMA*, *COL1A1*, *COLIII,* and *FN*. Moreover, knock down TAPA1 expression led to AITC losing its anti-TGF-β1 effects. AITC was administered 1 h prior to TGF-β1. * *p* < 0.05 vs. control; # *p* < 0.05 vs. TGF-β1. Data are shown as mean ± SEM and are from six experiments.

Since the administration of TRPA1 antagonist only partially blocked the anti-TGF-β1 effects of AITC, it was possible that the actions of AITC occurred though both TRPA1-dependent and TRPA1-independent pathways. To prove the involvement of TRPA1, we reduced the expression of TRPA1 channels in cultured subu−MyoFBs utilizing siRNA-mediated knockdown. TRPA1-siRNA treatment reduced the mRNA expression levels of *TRPA1* to 19.4% of that seen in the untreated cells; this was further reduced to 4.3% via TGF-β1 treatment (Figure 4A). We found that knocking down the expression of TRPA1 significantly enhanced the mRNA expression levels of *α-SMA*, collagens (type I and III), fibronectin, and TGF-β1 (Figure 4B–E), thus suggesting that TRPA1 exerts an anti-fibrotic effect in resting conditions (not stimulated). Furthermore, AITC lost anti-TGF-β1 effects in TRPA1-siRNA-treated myofibroblasts (Figure 4B–E).

### 2.5. TRPA1 Agonist Suppressed *SCI*-Induced Bladder Fibrotic Changes In Vivo

**Figure 5:**
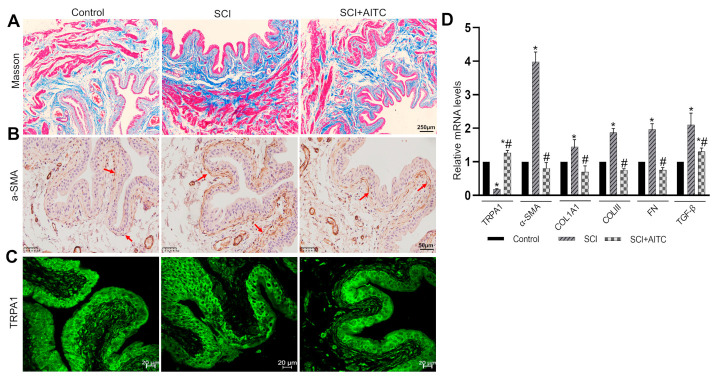
Activation of TRPA1 inhibited fibrotic changes in bladder mucosa from SCI rat. Rats were divided into the following three groups: control group (rats underwent sham surgery and received corn oil by gavage daily), SCI group (rats underwent SCI surgery and received corn oil by gavage daily), and SCI + AITC group (rats underwent SCI surgery and received AITC by gavage daily for 4 weeks starting on the 5th week). Eight weeks after surgery, rats were sacrificed for bladder histology and PCR experiments. (**A**) Masson’s staining showed that bladder from SCI rats exhibit increased collagen (blue) and that this effect could be partially attenuated by AITC treatment. Scale bar = 250 µm. (**B**) Immunohistochemical analysis of α-SMA expression (stained in brown) in the suburothelial layer showing increased α-SMA protein levels in SCI rats and the partial inhibition of this increase by AITC treatment. Scale bar = 50 µm. The red arrows indicate positive staining for α-SMA. (**C**) Immunofluorescence analysis of TRPA1 in the suburothelial layer showed that levels of TRPA1 protein were reduced in SCI rats and that this could be prevented by AITC treatment. Scale bar = 20 µm. (**D**) Analysis of the mRNA levels of *TRPA1*, *α-SMA*, *COL1A1*, *COLIII*, *FN,* and *TGF-β* in bladder mucosa from three groups of rats revealed similar findings to our in vitro experiments (Figure 3A–D). Data are shown as means ± SEM (*n* = 6 rats in each group); * *p* < 0.05 vs. control; # *p* < 0.05 vs. SCI.

To further investigate whether TRPA1 activation could counteract bladder fibrotic changes in vivo, a rat model of SCI was established. Consistent with previous reports [29], the bladders from SCI rats (8 weeks post-injury) exhibited significant deposition of collagen (Masson staining, Figure 5A) in the suburothelial layer. Immunostaining revealed the enhanced expression of α-SMA (Figure 5B) but reduced expression of TRPA1 (Figure 5C) in the suburothelial layer of the bladder when compared with the control rats. At the mRNA level, the SCI rats exhibited significantly increased expression levels of *α-SMA*, collagens (type I and III), fibronectin, and *TGF-β*, and a reduction in the expression levels of *TRPA1* in the bladder mucosa (Figure 5D). These SCI-induced fibrotic changes were attenuated in the rats that received AITC treatments by gavage daily for 4 weeks (Figure 5A–D) starting on the fifth week after the initial injury.

### 2.6. TRPA1 Was Down-Regulated in Fibrotic Human Bladder Mucosa

**Figure 6:**
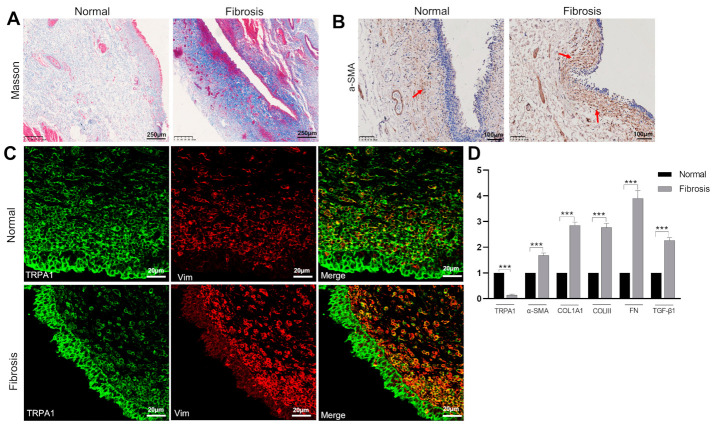
The expression levels of TRPA1 were decreased in the mucosa of human fibrotic bladders. (**A**) Masson’s staining of a human neurogenic bladder (right) showed significant collagen deposition (blue) in the suburothelial layer when compared to normal bladder (left). Scale bar = 250 µm. (**B**) Immunohistochemical analysis of α-SMA in the suburothelial layer indicating a significant increase in α-SMA expression in fibrotic bladder. Scale bar = 100 µm. The red arrows indicate positive staining for α-SMA. (**C**) Immunofluorescence analysis of TRPA1 (green) and vimentin (Vim, red) in the suburothelial layer showing reduced expression of TRPA1 in Vim-expressing cells in the submucosa of fibrotic human bladder when compared to normal human bladder. Scale bar = 20 µm. (**D**) Analysis of the mRNA levels of *TRPA1*, *α-SMA*, *COL1A1*, *COLIII*, *FN,* and *TGF-β1* in mucosa, indicating a significant increase in the expression of *α-SMA*, *COL1A1*, *COLIII*, *FN,* and *TGF-β1*, but a significant decrease in the expression of *TRPA1* in fibrotic bladder when compared with normal bladder. Data are shown as means ± SEM (*n* = 3 bladders in each group). *** *p* < 0.001.

To further investigate the role of TRPA1 in bladder fibrosis in vivo, we investigated the expression of TRPA1 and fibrotic changes in the bladder mucosa of patients with neurogenic bladders (*n* = 3) resulting from spinal cord injury. Urodynamic tests revealed that these patients had low compliance bladders. Masson staining revealed the dense deposition of collagen fibers (intense blue) in the suburothelial layer when compared with the normal bladder (Figure 6A). Immunohistochemistry revealed the enhanced protein expression of α-SMA (intense brown) in the suburothelial layer of the bladder in these patients (Figure 6B). Accordingly, we detected increased mRNA expression levels of *α-SMA*, collagens (type I and III), and fibronectin in samples of bladder mucosa from these patients (Figure 6D). However, TRPA1 expression was significantly reduced in these patients, both at the mRNA and protein level (Figure 6C,D).

## 3. Discussion

In our study, we investigated the role of TRPA1 channel activation on TGF-β1-induced fibrotic changes in subu−MyoFBs both in vitro and in vivo. The main findings can be summarized as follows: (1) in response to TGF-β1 stimulation mRNA and protein expression of α-SMA, collagen type I alpha 1 chain, collagen type III and fibronectin increased substantially in subu-MyoFB cells; (2) treatment with TRPA1 agonists suppressed these TGF-β1-induced fibrotic changes; (3) TGF-β1 down-regulated the expression of TRPA1 in cultured subu-MyoFB; (4) TRPA1 was down-regulated and α-SMA was up-regulated in the bladder mucosa of SCI rats. Additionally, these changes were partially reversed by AITC treatment; (5) the down-regulation of TRPA1 was also detected in the bladder mucosa of patients with neurogenic bladders. Our results suggest that TRPA1 expressing subu-MyoFB cells play an important role in the development of bladder fibrosis. It appears that the negative cross talk between TRPA1 and TGF-β1 signaling pathways may be a key mechanism in the pathogenesis of bladder fibrotic changes.

Immediately below the bladder urothelium, there is a layer of interstitial cells. Because these cells express the myofibroblast markers vimentin (Vim) and α-SMA, they are commonly termed suburothelial myofibroblasts (subu−MyoFBs) [21,30]. As interstitial cells, subu−MyoFBs have been the object of significant research over recent years; because these cells are located close to urothelial cells and sensory nerve terminals [31], they exhibit spontaneous electrical and Ca^2+^ activities [21,32] and express various receptors and channels that respond to neurotransmitters such as ATP and acetylcholine [21]. As a result, it was proposed that they play an important role in the transduction of sensory signals from the urothelium to the detrusor. Interestingly, a recent study reported that Vim^+^ ICs including subu−MyoFBs in the mouse bladder wall exhibit all of the ultrastructural hallmarks of fibroblasts and express multiple canonical as well as the universal fibroblast gene markers. Thus, it was proposed that these Vim^+^ ICs in the bladder wall are fibroblasts and may contribute to bladder remodeling and fibrosis [25]. Consistent with their proposal, we found that human subu−MyoFBs play roles in the formation of the ECM or bladder fibrosis. After TGF-β1 stimulation (in vitro), human subu−MyoFBs increased the gene and protein expression of (1) α-SMA, an important marker of active myofibroblasts; (2) collagens type I and III, both important components of ECM; and (3) fibronectin, an indicator of myofibroblasts (Figure 1 and Figure 3) [33]. Most importantly, even without TGF-β1 stimulation, these cells also expressed α-SMA, collagens, and fibronectins (Figure 1B). These myofibroblasts may be responsible for the production of ECM in normal bladder mucosa. Furthermore, it appears that under pathological conditions, such as spinal cord injury or longstanding BOO, these cells become more active and produce more ECM, resulting in fibrotic changes. Our findings also suggest that TGF-β1-induced fibrotic changes in subu−MyoFBs could serve as a model to study the mechanisms underlying bladder fibrosis.

In the bladder, TRPA1 is mainly expressed in a proportion of TRPV1-expressing sensory nerves and in human urothelial cells [34]. These channels were implicated in both pain perception and the modulation of detrusor mobility [35,36]. Previous studies from our laboratory and others indicate that most (>90%) subu−MyoFBs in humans, guinea pigs, and pigs express TRPA1 channels at the mRNA, protein, or functional level [26,27]. However, the specific functions of TRPA1 in myofibroblasts have yet to be elucidated. In this study, we found that TRPA1 agonists could counteract TGF-β1-induced fibrotic changes (Figure 3), thus suggesting an anti-fibrotic role for TRPA1 channels. This suggestion concurs with previous reports in hepatic stellate cells [14], the oral submucosa [15], the lungs [16], and the intestine [17,18,19]. However, this suggestion does not concur with other studies carried out in the heart and corneal stroma, which showed that the inhibition of TRPA1 results in anti-fibrosis effects [37,38]. The reasons for such inconsistency are not yet clear. These observations may suggest that the regulatory role of TRPA1 in tissue fibrosis may be organ dependent.

The anti-fibrotic effects of TRPA1 agonists may be TRPA1-dependent or TRPA1-independent [39]. Our current findings demonstrate that a TRPA1 antagonist (HC030031) only partially blocked the anti-fibrotic effects of AITC, thus suggesting that both mechanisms were involved. However, there are several lines of evidence to support the fact that TRPA1 activation may play a prominent role in the following: (1) knocking down TRPA1 expression reduced TGF-β1-induced fibrotic changes (Figure 4); (2) the anti-fibrotic effects of TRPA1 agonists only occurred when AITC was applied prior to TGF-β1 treatment (i.e., after TRPA1 activation), but not when AITC was applied together with or after TGF-β1, where TRPA1 was already strongly inhibited by TGF-β1 and very few TRPA1 channels were available for TRPA1 agonists and antagonists (Figure 3). The downregulation of TRPA1 by TGF-β1 may help to explain why the TRPA1 antagonist could not completely block TGF-β1-induced fibrotic changes in fibroblasts in the intestine or lungs [4,16,40]. Although our findings indicate that the activation of TRPA1 predominantly mediated the anti-fibrotic effects of TRPA1 agonists, we cannot exclude TRPA1-independent mechanisms. AITC was shown to inhibit FMT through TRPA1-independent ERK/MAPK and NRF2/HO-1 pathways in lung fibroblasts [16].

The canonical signal pathway of TGF-β1 involves Smad2/3 phosphorylation [28]. We found that AITC significantly attenuated TGF-β1-induced Smad2 phosphorylation (Figure 3E). The exact mechanisms underlying the inhibition of Smad2 phosphorylation remain unclear, but may involve the activation of TRPA1-induced Ca^2+^ influx because the influx of Ca^2+^ is known to activate PKA; the cAMP/PKA pathway was shown to inhibit the Smad signaling pathway in lung fibroblasts [40].

In addition to the counteracting effect of TRPA1 on TGF-β1, we found that TGF-β1 exerted strong inhibition on the expression of TRPA1 (Figure 2E,F). In fibrosis, one scenario is that increased TGF-β1 may stimulate myofibroblasts to produce more products of oxidative stress (ROS) such as H_2_O_2_, which may, in turn, activate latent TGF-β1, thus developing a profibrogenic cycle [16,41]. Another situation is that the persistent increased production of H_2_O_2_ [42] could activate TRPA1 to induce Ca^2+^ overload and the death of myofibroblasts. The persistent presence of myofibroblasts is one of the most important mechanisms underlying fibrogenesis. It is not yet clear how myofibroblasts can survive in fibrosis conditions. In lung myofibroblasts, the TGF-β1-induced down-regulation of TRPA1 was found to be a dominant mechanism for the resistance of lung myofibroblasts to ROS [39]. In the present study, the TGF-β1-induced downregulation of TRPA1 was observed in cultured subu−MyoFBs (Figure 2E,F), in the bladder mucosa of SCI rats (Figure 5C,D) as well as in the mucosa of neurogenic bladder patients (Figure 6C,D). Thus, therapies that target this mechanism may have the potential to prevent fibrosis progression and perhaps even reverse established bladder fibrosis, as suggested by studies in other tissues [43,44].

There seems to be a positive TRPA1 staining in the urothelium from the rat (Figure 5C) and human bladder (Figure 6C). The expression of TRPs in urothelium is debatable as extensively discussed [45]. There are reports in favor of TRPA1 expression, but more recent papers demonstrating the initial reports were incorrect [45]. It is thought that the positive staining revealed with immunohistochemistry or immunofluorescence methods does not mean that the channels are functional. In support of this idea, our previous study with calcium imaging showed that the TRPA1 agonist AITC (100 µM) did not evoke a significant increase in [Ca^2+^]_i_ in the urothelial cells, suggesting no functional expression [27].

Our study has some limitations. First, the specific underlying mechanism of TRPA1-regulating bladder fibrosis was not explored in depth. Further investigations should be focused on the downstream signal pathways involved in TRPA1 anti-fibrotic action. Only the blocking peptide was used to validate the specificity of the TRPA1 antibody in our study; other approaches such as TRPA1-knockout rats should be used to further confirm its specificity. In addition, given that the siRNA-mediated knockdown failed to completely eliminate TRPA1 expression, TRPA1-knockout rats should be generated and tested to further confirm the role of TRPA1 agonists in bladder fibrosis.

## 4. Materials and Methods

### 4.1. Ethical Approval of the Study Protocol

Bladder tissue samples for the culture of subu−MyoFBs were obtained from four patients (one female, three males; mean age: 57.6 ± 14.1 years) undergoing cystectomy for bladder carcinoma. Fibrotic bladder tissues were obtained from three male patients (ages 32–50 years) undergoing bladder augmentation surgery because of neurogenic lesions. The use of these samples was approved by the ethics committee of Second Hospital, Shandong University (KYLL-2022LW139), and all patients provided written consent for the use of their tissues.

Female Sprague Dawley rats (2–3-month-old virgins, weighing 180–250 g) were purchased from Wugyue Animal Company (Jinan, China). Animal care and handling were carried out in accordance with the guidance of the Shandong University Animal Care and Use Committee. The animal study was approved by the Ethics Committee of the Second Hospital, Shandong University (KYLL-2020kJA-0074).

### 4.2. Human Suburothelial Myofibroblasts Cultures

Subu-MyoFBs were cultured as described previously [27]. Briefly, tumor-free bladder mucosa was separated from the detrusor layer. Small fragments (~1 mm^2^) of this tissue were digested with trypsin at 37 °C for 15 min. After terminating the enzyme activity by the application of 10% fetal bovine serum, the resulting cell suspension was plated into tissue culture flasks and incubated in an atmosphere of 5% CO_2_ at 37 °C. Smooth Muscle Cell Growth Medium 2 (Procell, Wuhan, China) was used as the culture medium to limit the growth of urothelial cells. Cells from passage-2 to passage-8 were used for experiments. The identity of the cells was confirmed by staining with the widely used interstitial cell markers Vim and α-SMA.

### 4.3. RT-qPCR and RT-PCR

When cultures reached >90% confluence in 6-well plates, cells were harvested by 0.25% trypsin treatment. Total RNA was then extracted using an RNA Simple Total RNA kit (Tiangen, Beijing, China). The concentration of RNA was determined via UV spectrophotometry and reverse transcription was carried out using the SPARKscript II RT plus Mix kit (Sparkjade, Qingdao, China) in accordance with the manufacturer’s instructions. The cDNA was amplified using the following cycling conditions: 40 cycles of denaturation for 15 s at 95 °C, annealing and elongation for 30 s at 60 °C. RT-qPCR experiments were conducted using SYBR TM Green qPCR Mix (Sparkjade) and an QuantStudio TM 5 system (Thermo Fisher, Waltham, MA, USA). Gene-specific primers were synthesized via BioSune (Shanghai, China), and the sequences of the used primer pairs are shown in Table 1. mRNA expression was quantitated using the 2^−ΔΔCt^ method.

For RT-PCR, total RNA was extracted from the cultured cells using TRIzol (Invitrogen, Carlsbad, CA, USA) and a DNA-free kit (Ambion, Foster City, CA, USA). cDNA was then synthesized via Superscript (Invitrogen) and the PCR was performed using Surestart Taq polymerase (Sparkjade).

### 4.4. Western Blotting

Cultured cells were lysed in RIPA lysis buffer (Beyotime, Shanghai, China) supplemented with a complete protease inhibitor cocktail (Beyotime, Shanghai, China) for 30 min on ice. After centrifugation (12,000× *g*, 4 °C, 15 min), the supernatants were collected and the protein concentration was measured using a Bio-Rad DC Protein Assay Kit (Bio-Rad, Hercules, CA, USA). Samples were mixed with a loading buffer and heated to 95 °C for 5 min, and 20 micrograms of total protein was loaded per lane on an SDS-PAGE gel. After electrophoresis, the proteins were transferred to polyvinylidene difluoride (PVDF) membranes (Merck Millipore, Darmstadt, Germany) at a constant current of 200 mA using the wet transfer method, adjusting the required transfer time according to protein molecular weight. The 10× Transfer buffer contained 116 g Tris base and 24 g glycine in 1 L of double distilled water; the 1× Transfer buffer consisted of 700 mL double distilled water, 200 mL methanol, and 100 mL 10× Transfer buffer. After blocking with 5% skimmed milk (dissolved in Tris-buffered saline) at room temperature for 2 h, the membranes were incubated overnight at 4 °C with the primary antibodies reacting with the following proteins: TRPA1 (Alomone, Jerusalem, Israel, ACC-037, 1:200); α-SMA (Abcam, Cambridge, UK, ab124964, 1:100); fibronectin (PTG, Wuhan, China, 15613-1-AP, 1:500); collagen type I (PTG, 66761-1-lg, 1:500); collagen type III (PTG, 22734-1-AP, 1:500); and glyceraldehyde-3-phosphate dehydrogenase (GAPDH) (Beyotime, AG019, 1:1000). After incubating with horseradish peroxidase-conjugated secondary antibodies (Zhongshan Co., Beijing, China, ZB-2301, ZB2305, 1:5000), antibody–antigen complexes were detected using an ECL substrate (Millipore, Billerica, MA, USA) and visualized using an Image Quant Tanon 4800 System (Beijing, China).

### 4.5. Immunofluorescence Staining of Cultured Cells

Subu-MyoFBs were cultured on coverslips and fixed with 4% paraformaldehyde for 15 min. Cells were blocked with 5% normal goat serum for 30 min and incubated overnight at 4 °C with shaking, using the primary antibodies (the same antibodies described in the Western blotting section, at dilution of 1:100). The specificity of antibodies used in our study was previously confirmed by the manufacturer and validated by other investigators [46]. For the TRPA1 antibody, pretreatment with its blocking peptide (Alomone, Jerusalem, Israel, BLP-CC037, 1:200) served as a negative control to confirm the specificity of the TRPA1 antibody. Subsequently, the coverslips were washed with PBS and incubated with the appropriate secondary antibodies for 1 h at room temperature. Staining was captured using a laser scanning confocal microscope (Zeiss) and analyzed via ImageJ software.

### 4.6. Intracellular Ca^2+^-Flux Imaging

Subu-myoFBs were cultured on glass coverslips and loaded with Fura-2-acetoxymethyl ester (Fura 2-AM; 2 µM; Dojindo Laboratories, Tongren, Japan) for 30 min. Fura 2-AM was dissolved in Hank’s balanced salt solution containing 138 mM NaCl, 5 mM KCl, 0.3 mM KH_2_PO_4_, 4 mM NaHCO_3_, 2 mM CaCl_2_, 1 mM MgCl_2_, 10 mM HEPES, and 5.6 mM glucose, at pH 7.4. Ca^2+^ imaging was carried out as described previously [47]. Briefly, coverslips were placed in a recording chamber, and the Fura 2-AM dye was excited via UV light alternatingly at 340 nm and 380 nm. Wavelength selection, the timing of excitation, and image acquisition were controlled using a MetaFluor device (Molecular Devices, Sunnyvale, CA, USA). The ratio of the fluorescence signal measured at 340 nm and 380 nm was used to measure the increase in [Ca^2+^]_i_. An increase in [Ca^2+^]_i_ was considered significant if the ratio change was >0.1.

### 4.7. TRPA1 RNA Interference

The expression of TRPA1 in subu−MyoFBs was inhibited using small interfering RNAs (siRNAs). Gene-specific siRNAs, along with the scrambled siRNA, were designed and synthesized by GenePharma (Shanghai, China). The *TRPA1* siRNA final sequences were 5′-UGGGAUGUUAUUCCAUAUAUTT (sense) and 5′-AUAUAUGGAAUAACAUCCCACC (antisense). The scrambled siRNA sequences were 5′-UUCUCCGAACGUGUCACGUtt-3′ (sense) and 5′-ACGUGACACGUUCGGAGAAtt-3′ (antisense).

Subu-MyoFBs were grown to 70–80% confluence and then incubated in Lipofectamine 2000 transfection reagent containing siRNA or scrambled siRNA for 12 h. Transfection efficiency was evaluated via mRNA and protein expression analysis using real-time qPCR and TRPA1 immunofluorescence, respectively.

### 4.8. Rat Model of SCI

Eighteen female Sprague Dawley rats were randomly assigned to three groups. The control group (*n* = 6) underwent sham surgery and received corn oil, the SCI group (*n* = 6) underwent SCI surgery and received corn oil, and the SCI + AITC group (*n* = 6) underwent SCI surgery and received AITC at 100 mg/Kg/d. The administration of corn oil (control substance) or AITC by daily gavage was started on the 5th postoperative week and continued for 4 weeks. A modification of Allen’s weight-drop technique was used to create moderate SCI. The incision site was determined by locating the 13th thoracic vertebra containing the floating ribs. A medial 3 cm incision was made on the back, extending into the skin and subcutaneous fascia. The paravertebral muscles were mobilized bilaterally via blunt dissection. The T8 spinous process was excised, and the T8 lamina was removed to expose the spinal epidural layer. A 6 g cylindrical weight was dropped from a 10 cm height through a hollow glass tube (2 mm diameter) onto the exposed spinal cord. Muscle and skin incisions were sutured layer-by-layer. Spinal injury was confirmed by bilateral hindlimb twitching and tail flicking, followed by complete muscle paralysis and mopping on the ground after the cessation of anesthesia. To prevent postoperative infections, animals received an intramuscular injection of gentamicin (5 mg/kg). Bladder tissues were removed for histological examination and RT-qPCR evaluation 8 weeks after the surgery.

### 4.9. Histological Staining

Rat and human bladder samples were fixed in 4% paraformaldehyde. After embedding in paraffin, 4μm tissue slices were prepared for Masson’s trichrome staining, immunohistochemistry (IHC), and immunofluorescence (IFC). Before IHC and IFC staining, paraffin-embedded sections were dewaxed, rehydrated, and incubated overnight at 4 °C using the following primary antibodies: anti-α-SMA (catalog ab7817, Abcam) or anti-TRPA1 (ACC-037, Alomone), both mixed with an anti-Vimentin (Vim, MA1-06908, Invitrogen) antibody. Then, sections for IHC were visualized via DAB. Sections for IFC were incubated with a goat anti-rabbit IgG (H + L) secondary antibody (proteintech, SA00013-2) or goat anti-mouse IgG (H + L) secondary antibody (ABclonal, AS077), treated with diaminobenzidine, and counterstained with hematoxylin or 40,6-diamidino-2-phenylindole (DAPI). IHC images were captured with a digital scanning microscope (Leica DM2000), while IFC images were obtained using a laser scanning confocal microscope (Zeiss). Masson’s trichrome staining, used to detect collagen deposition, was conducted in accordance with standard protocols (Solarbio). This protocol stains muscle fibers blue, and stains nuclei black, while the cytoplasm is stained red.

### 4.10. Other Reagents

The following additional reagents were used in this study: TGF-β1 (Peprotech, New Jersey, USA), Allylisothiocyanate (AITC, Sigma-Aldrich Inc., Darmstadt, Germany), cinnamaldehyde (CA, Selleck, TX, USA), and HC030031 (Abcam, Cambridge, UK). Stock solutions of TGF-β1 were prepared in citric acid, while AITC, CA, and HC030031 were dissolved in DMSO. The final concentration of DMSO used in tissue culture and animal experiments was <0.1%. All the above reagents were used at concentrations based on previous reports in the literature.

### 4.11. Statistical Analyses

Data are presented as means ± standard error of the mean (SEM) and were analyzed using SigmaPlot 14.0 (Systat Software Inc., San Jose, CA, USA) and GraphPad Prism 8.0 statistical software (GraphPad software). Statistical significance was determined via one- or two-way analysis of variance (ANOVA), followed by Holm–Sidak analysis for the comparison of multiple groups, and Student’s t-test when comparing two groups. *p* < 0.05 was considered statistically significant.

## 5. Conclusions

In summary, our study generated three important findings. First, bladder subu−MyoFBs can be considered as myofibroblasts cells from a functional perspective. Second, there is a negative cross talk between TRPA1 and TGF-β1 signaling in bladder subu−MyoFBs. Third, the downregulation of TRPA1 may be an important survival mechanism for myofibroblasts. Our findings may provide a new option for interpreting the theory of bladder fibrosis pathogenesis and may have notable therapeutic significance, given that the enhancement of the TGF-β1 signal pathway has been implicated in various models of bladder fibrosis. The mechanisms by which TGF-β1 can induce the downregulation of TRPA1 may play critical roles in the persistence of myofibroblasts and bladder fibrosis. Strategies that activate TRPA1 or prevent the TGF-β1-induced downregulation of TRPA1 may be able to prevent or reverse bladder fibrosis.

## Figures and Tables

**Table 1:** Oligonucleotide primer sets for quantitative real-time PCR (RT-qPCR).

Name	Sequence (5′–3′)	Length	Tm
*Rat-ACTA2 F*	ACCATCGGGAATGAACGCTT	20	60
*Rat-ACTA2 R*	CTGTCAGCAATGCCTGGGTA	20	60
*Rat-COL1A1 F*	CACTGCAAGAACAGCGTAGC	20	60
*Rat-COL1A1 R*	AAGTTCCGGTGTGACTCGTG	20	60
*Rat-COLIII F*	CAGCCTTCTACACCTGCTCC	20	62
*Rat-COLIII R*	GTCGCCATTTCTCCCAGGAA	20	62
*Rat-TRPA1 F*	CCATGGGTGGCTACACTCAG	20	62
*Rat-TRPA1 R*	TCAAAAGCATCGCAACAGCC	20	62
*Rat-FN F*	AAGCTACCATTCCAGGCCAC	20	62
*Rat-FN R*	GTCACTTCTTGGTGCCCGTA	20	62
*Rat-β-actin F*	CTCTGTGTGGATTGGTGGCT	20	62
*Rat-β-actin R*	CGCAGCTCAGTAACAGTCCG	20	62
*Homo-ACTA2 F*	ATGCCTCTGGACGCACAACT	20	62
*Homo-ACTA2 R*	CCCGGACAATCTCACGCTCA	20	62
*Homo-TRPA1 F*	CAGAAGACAAGTCCTGCCGA	20	60
*Homo-TRPA1 R*	TTGAGGGCTGTAAGCGGTTC	20	60
*Homo-COL1A1 F*	GCCAAGACGAAGACATCCCA	20	60
*Homo-COL1A1 R*	GGCAGTTCTTGGTCTCGTCA	20	60
*Homo-COLIII F*	CTGGTCCCGAAGGAGGAAAG	20	59
*Homo-COLIII R*	TAGGACCAGTAGGACCCCTTG	20	59
*Homo-FN F*	TGTGAACATCCCTGACCTGC	20	60
*Homo-FN R*	CAGGCGCTGTTGTTTGTGAA	20	60
*Homo-TGFβ1 F*	GCAACAATTCCTGGCGATACC	21	60
*Homo- TGFβ1 R*	ATTTCCCCTCCACGGCTCAA	20	60
*Homo-β-Actin F*	CATGTACGTTGCTATCCAGGC	21	57.6
*Homo-β-Actin R*	CTCCTTAATGTCACGCACGAT	21	55.6

## Data Availability

Data will be made available upon request.

## Abbreviations

AITC	allyl isothiocyanate
α-SMA	alpha-smooth muscle actin
BOO	bladder outflow tract obstruction
ECM	extracellular matrix
SCI	spinal cord injury
Subu-MyoFBs	suburothelial myofibroblasts
TGF-β1	transforming growth factor-1
TRP	transient receptor potential
